# Biofabrication of Collagen Tissue-Engineered Blood Vessels with Direct Co-Axial Extrusion

**DOI:** 10.3390/ijms23105618

**Published:** 2022-05-17

**Authors:** Èlia Bosch-Rué, Leire Díez-Tercero, Luis M. Delgado, Román A. Pérez

**Affiliations:** 1 Bioengineering Institute of Technology (BIT), Universitat Internacional de Catalunya (UIC), Sant Cugat del Vallès, 08195 Barcelona, Spain; ebosch@uic.es (È.B.-R.); ldiezter@uic.es (L.D.-T.); lmdelgado@uic.es (L.M.D.); 2 Basic Sciences Department, Universitat Internacional de Catalunya (UIC), Sant Cugat del Vallès, 08195 Barcelona, Spain

**Keywords:** tissue-engineered blood vessels, collagen, co-axial extrusion, vascular construct, cell-laden hydrogels

## Abstract

Cardiovascular diseases are considered one of the worldwide causes of death, with atherosclerosis being the most predominant. Nowadays, the gold standard treatment is blood vessel replacement by bypass surgery; however, autologous source is not always possible. Thereby, tissue-engineered blood vessels (TEBVs) are emerging as a potential alternative source. In terms of composition, collagen has been selected in many occasions to develop TEBVs as it is one of the main extracellular matrix components of arteries. However, it requires specific support or additional processing to maintain the tubular structure and appropriate mechanical properties. Here, we present a method to develop support-free collagen TEBVs with co-axial extrusion in a one-step procedure with high concentrated collagen. The highest concentration of collagen of 20 mg/mL presented a burst pressure of 619.55 ± 48.77 mmHg, being able to withstand perfusion of 10 dynes/cm^2^. Viability results showed a high percentage of viability (86.1 and 85.8% with 10 and 20 mg/mL, respectively) of human aortic smooth muscle cells (HASMCs) and human umbilical vein endothelial cells (HUVEC) after 24 h extrusion. Additionally, HUVEC and HASMCs were mainly localized in their respective layers, mimicking the native distribution. All in all, this approach allows the direct extrusion of collagen TEBVs in a one-step procedure with enough mechanical properties to be perfused.

## 1. Introduction

Cardiovascular diseases (CVDs) are one of the leading causes of death according to the World Health Organization (WHO), representing 32% of all global deaths [1]. One of the main causes of CVDs is atherosclerosis, consisting of a buildup of fatty plaque in the inner wall of blood vessels which eventually narrows the blood flow leading to tissue ischemia [2]. Nowadays, the gold standard treatment for atherosclerosis consists of the use of stents or blood vessel replacement by bypass surgery. However, the use of the stents in clinical practice still has some in-stent restenosis concerns [3], and continues to be a problem in clinical practice [4]. The use of autologous grafts such as the saphenous vein or mammary artery is also another strategy used in clinics [5,6], but their use for blood vessel replacement is not always possible due to the previous harvest or the inherent illness of the patient. In this regard, the development of tissue-engineered blood vessels (TEBVs) as alternative grafts for bypass surgery is an area of extensive research [7]. Different methods have been described for the development of TEBVs, such as sheet rolling, direct scaffolding using electrospinning, matrix molding or even decellularization [8]. However, these approaches involve multiple steps in order to obtain a vascular construct seeded with vascular cells.

An ideal TEBV should provide cells with an extracellular matrix (ECM) similar to that of native tissue, mainly collagen, while allowing cells to spread throughout the structure in low periods of time to provide the required functionality to the TEBV [9]. Generally, when collagen is used for TEBV manufacturing, some support or additional processing is necessary to avoid its spreading and to maintain tubular shape while being gelled. In this regard, matrix molding (also known as casting) is the method of choice [10,11,12]. However, additional steps are required to increase the mechanical stability of these TEBVs, for instance, drying the vascular conduit overnight at 37 °C to increase collagen density and subsequently cross-link it with genipin [11], or by plastic compression [12]. In the last few years, direct co-axial extrusion using core-shell nozzles has gained special attention due to the ability to develop a multiple layer construct, considerably reducing the manufacturing process in a single fabrication step [13]. At the same time, this method proved to be successful for the extrusion of cells without compromising their viability and proliferative capacity. In order to develop collagen TEBVs with co-axial extrusion, there is the need to combine it with other biomaterials with instantly crosslinking properties to provide structural support when being extruded, as collagen itself is not self-supportive [14,15]. In this regard, the most used is alginate due to its instant gelling ability when in contact with divalent ions [16]. In this sense, we developed a core shell TEBV presenting alginate in the outer layer to provide structural support, together with collagen in the inner layer. Our results showed that we could extrude endothelial cells in the inner layer and smooth muscle cells in the outer layer, with a parallel and concentric alignment, respectively, similar to native vascular cell organization [17]. Nevertheless, the higher amounts of alginate compared to collagen limited the adhesion, proliferation and initial cell stretching of the smooth muscle cells (SMC) [17].

Here, we aim to develop tissue-engineered blood vessel (TEBV)-like structures by using high concentrations of collagen both in the inner and outer layer through co-axial extrusion method to allow encapsulating SMC and human umbilical vein endothelial cells (HUVEC) in two distinct layers, both being collagen-based. To this purpose, we will assess the ability of the collagen concentrations to provide a self-supporting structure. The cell survival ability of both cell types after extrusion will be tested in their respective vascular layers while maintaining the initial allocation. To the best of our knowledge, the direct extrusion of solely collagen with vascular cells for TEBV development has not been reported before with the co-axial extrusion method.

## 2. Results

### 2.1. Optimal Conditions for High Concentration Collagen TEBV Extrusion

Both 10 and 20 mg/mL of collagen could be dissolved, obtaining a homogenous viscous solution with a gelling time of 10–15 min at 37 °C. As a first step, the choice of the extrusion bath for the extrusion of collagen TEBV was studied. Then, the effect of the injection speed on the size of TEBVS and which one facilitated the extrusion process was investigated.

**Figure 1:**
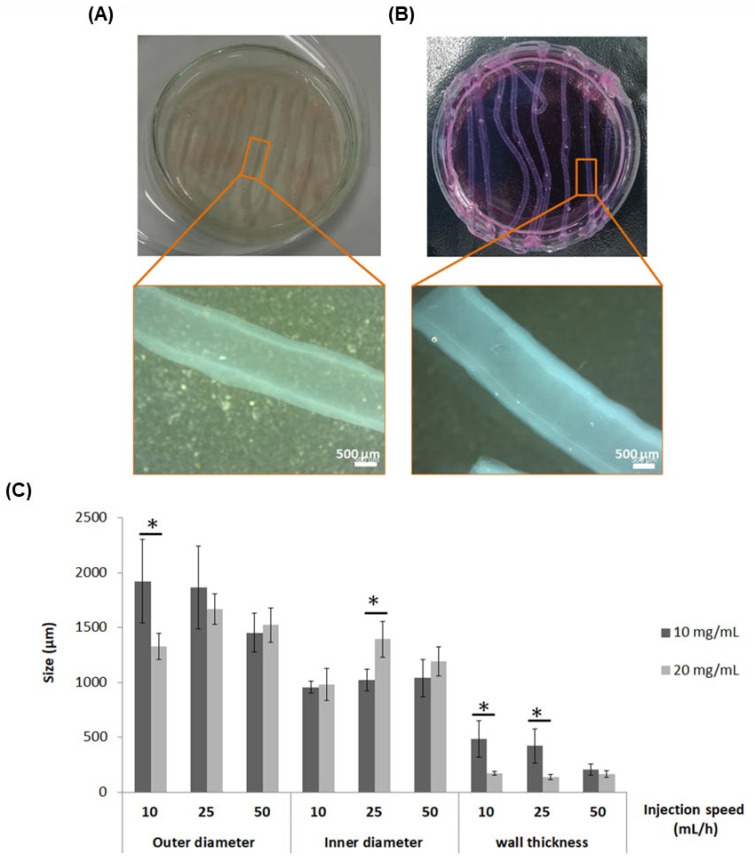
Tissue-engineered blood vessel (TEBV) support bath extrusion optimization and TEBV sizes. TEBVs extruded with collagen concentration of 10 mg/mL in (**A**) gelatin microparticles or (**B**) in cell culture media, with 25 mL/h as the injection speed. (**C**) Size of outer diameter, inner diameter and wall thickness depending on the injection speed with collagen at 10 mg/mL and 20 mg/mL. Data represented as mean with standard deviation (*n* = 8 per condition). * Statistically significant differences between the two collagen concentrations for the same injection speed (*p* < 0.05).

On the one hand, regarding the extrusion baths, gelatin microparticles size was around 100 µm, as illustrated in Supplementary Figure S1. The gelatin microparticle slurry allowed the extrusion of TEBVs maintaining their tubular structure (Figure 1A). More interestingly, both concentrations of collagen provided self-stability to allow the extrusion of TEBV in cell culture media while preserving the tubular structure without spreading, as observed in Figure 1B. Taking into account that vascular cells would be incorporated within the TEBV structure, the use of cell culture media as the extrusion bath was selected for the subsequent assays.

On the other hand, the results of TEBVs sizes (outer diameter, inner diameter and wall thickness) depending on the injection speed and collagen concentration are represented in Figure 1C. In general, the effect of injection speed on the size depended on the collagen concentration. The results showed that as the injection speed was increased, the size of the TEBV (outer diameter) was reduced with the 10 mg/mL concentration, while it was increased with the 20 mg/mL collagen concentration, although the differences were not significant. Nonetheless, the inner diameter remained the same with 10 mg/mL for all injection speeds tested, whereas it was increased with 20 mg/mL. It is worth highlighting that the wall thickness was significantly higher with the 10 mg/mL collagen concentration, especially for the injection speeds of 10 and 25 mL/h, with a thickness around 500 µm, whereas it was around 200 µm for the collagen concentration of 20 mg/mL. Additionally, the coefficient of variation (CV) for the wall thickness with the 10 mg/mL for the injection speeds of 10, 25 and 50 mL/h were 34.5, 36.8 and 25.7%, respectively. With the 20 mg/mL, the CV were 10.8, 17.1 and 17.9% for 10, 25 and 50 mL/h, respectively, suggesting a more homogenous wall thickness with the highest collagen concentration.

As the triple co-axial nozzle had to be moved manually along the petri dish during extrusion, the 25 mL/h injection speed was selected for subsequent experiments. This injection speed allowed more uniform control of the movement of the triple co-axial nozzle, facilitating the formation of straight TEBVs, whereas with 50 mL/h, this fact was not possible, and for the 10 mL/h, the time needed to extrude the TEBV was significantly increased.

### 2.2. Mechanical Properties and Preliminary Results of Tissue-Engineered Blood Vessels (TEBVs) Perfusion

Burst pressure was measured, as previously described, by the infusion of PBS inside acellular TEBV until failure. Results demonstrated that 20 mg/mL collagen TEBVs could withstand up to 619.55 ± 48.77 mmHg. Moreover, as a proof of concept, we attached these acellular TEBVs in a perfusion chamber and perfused them with cell culture media at 37 °C up to 3 days with a physiological flow rate equivalent to arterial shear stress of 10 dynes/cm^2^. These TEBVs demonstrated their ability to withstand physiological mechanical stimulation without rupture (Supplementary Video S1).

### 2.3. Viability of HUVEC and HASMC Extruded within TEBVs

**Figure 2:**
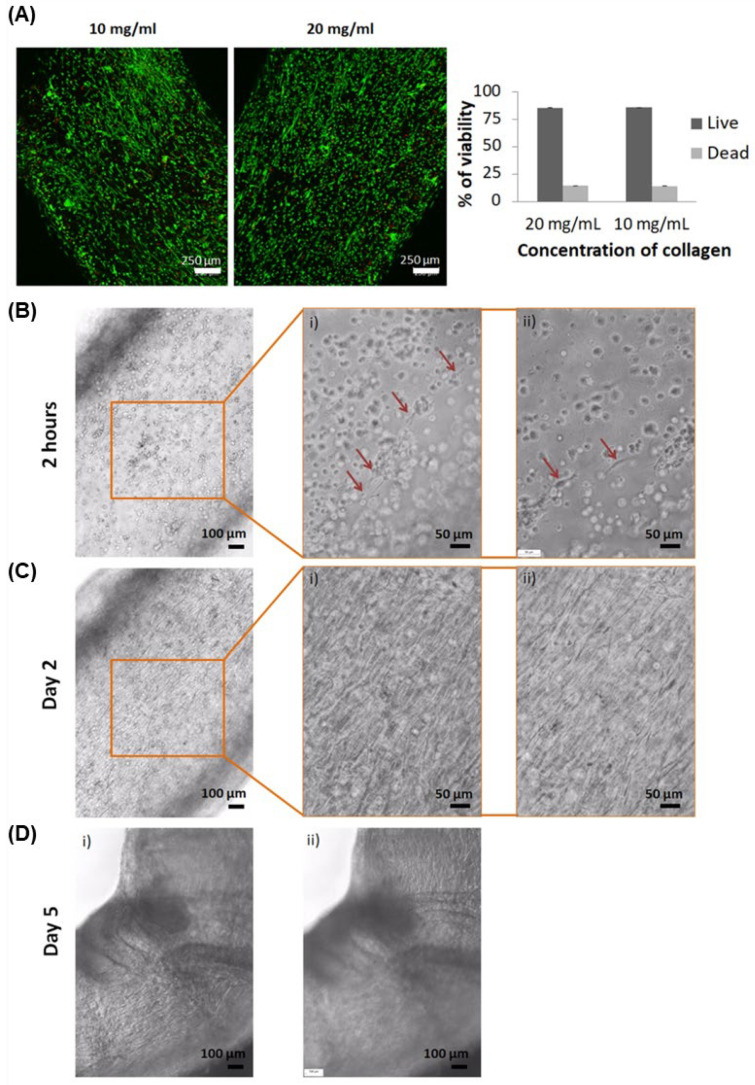
Viability and images of TEBVs with human umbilical vein endothelial cells (HUVEC) and human aortic smooth muscle cells (HASMC) in co-culture. (**A**) Live/dead assay performed on TEBV containing HUVEC and HASMCs in co-culture at 24 h after extrusion (live cells in green and dead cells in red). (**B**) First cells spreading after 2 h of TEBV culture. On the right are presented magnifications at two different plans with red arrows indicating stretched cells. (**C**) Practically all cells were lengthened within 2 days of TEBV culture. On the right are magnifications at two different plans. (**D**) TEBVs started to blend from day 5 at different focus, showing cells on top (i) and cells at the bottom of the TEBV (ii). Images from two different plans.

Cell viability was assessed 24 h after the extrusion process, resulting in 86.1 ± 0.02% and 85.8 ± 0.01% of live cells for 10 and 20 mg/mL of collagen, respectively (Figure 2A). At this point, as both concentrations presented similar high viability, the highest concentration (20 mg/mL) of collagen was selected for the subsequent assays, as it would provide higher mechanical stability, hence allowing more robust manipulation (Supplementary Video S2).

During cell culture of TEBVs with 20 mg/mL of collagen, some cells started to proliferate within few hours, as shown in Figure 2B. After 2 days, practically all cells were found stretching (Figure 2C), and from day 5, TEBVs started to fold (Figure 2D) due to cell stretching. Each cell type could not be distinguished during culture time. However, these observations were consistent throughout all TEBV 3D structure plans.

### 2.4. The 3D Reconstruction of Collagen TEBV

**Figure 3:**
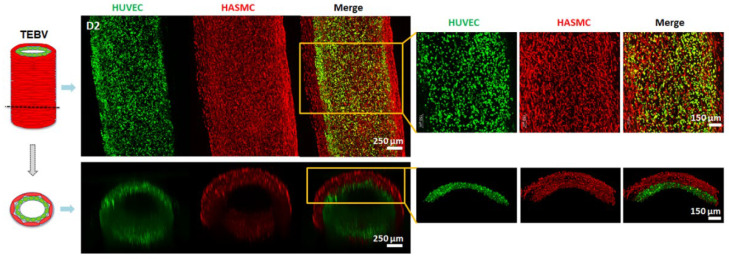
TEBV cell tracker reconstruction with confocal microscope at day 2. Each color indicates different cell types: HUVEC in green and HASMCs in red at day 2. On the right there is a magnification of the TEBV.

HUVEC and HASMCs were stained with specific cell tracker in green and red color, respectively, in order to visualize their distribution and to assess whether cells were allocated in their respective layers within the TEBV construct. As can be observed in Figure 3, cells covered the tubular structure within 2 days of culture. It is worth mentioning that very few cells were localized in locations outside their defined layer.

## 3. Discussion

An important consideration to take into account for TEBV development is the mimicking of the extracellular matrix composition [18]. In this study, we aimed to extrude TEBVs with high concentration of collagen type I as the unique biomaterial composition, to assess if vascular cells were able to survive within them and if they had enough mechanical properties to withstand perfusion equivalent to arterial shear stress. To this purpose, we first tested two different support baths for their extrusion, to determine if they required structural support to maintain their tubular structure once extruded. One of them was gelatin microparticles, based on a previous study [19], to avoid its spreading after its extrusion. The gelatin microparticle size reported in this study was approximately 25 µm, whereas we obtained a microparticle size around 100 µm (Supplementary Figure S1). Nonetheless, the size we obtained allowed us to extrude collagen TEBVs, while maintaining their tubular structure (Figure 1A). Remarkably, the high concentrations of collagen tested also allowed the tubular structure to be maintained when directly extruded in cell culture media solution without spreading (Figure 1B), demonstrating that no structural support was needed to extrude TEBVs. Previous studies developing TEBVs made of solely collagen required the use of a structural support to maintain the collagen tubular structure while gelling, generally using the matrix molding or casting approach [10,11,12]. Here, we demonstrated that TEBVs could be directly extruded in cell culture media by just increasing the concentration of collagen type I, requiring a unique one-step procedure for their development, avoiding the need to previously fabricate a support bath. Additionally, we considered that the presence of nutrients and growth factors within cell culture media would ameliorate cell survival for future vascular cell encapsulation.

Regarding the sizes of TEBVs, interesting results were obtained when using different collagen concentrations. More specifically, wall thickness was in general higher when 10 mg/mL was used compared to 20 mg/mL (Figure 1C). Lower collagen concentration tested had lower consistency, and therefore, during the transition from liquid to gel state, possible spreading could take place resulting in a higher wall thickness. This reason might also explain thinner wall thickness of TEBVs when 20 mg/mL was used, presenting a lower coefficient of variation, which was indicative of more homogeneous wall size. In general, there were no big differences in outer and inner diameter TEBV size with the different injection speeds for each collagen concentration. However, between the three injection speeds tested, 25 mL/h was the one which facilitated the most uniform movement of the triple co-axial nozzle while extruding. The sizes of these TEBVs were similar to TEBVs developed in a previous study using the same triple co-axial nozzle but using different biomaterials (alginate and collagen) [17], with the outer and inner diameters being around 1600 and 1400 µm, which corresponds to the range of arteries, as described, to be within 100 µm and 10 mm [20]. However, only the 20 mg/mL had a similar wall thickness of approximately 200 µm, described to be the limit to allow nutrient and oxygen diffusion [21,22].

Another important characteristic for the development of TEBV is to possess enough mechanical properties to be perfused [23]. In this study, we obtained a burst pressure of approximately 620 mmHg for the 20 mg/mL collagen TEBVs. One of the first studies developing TEBVs made of collagen reported a burst pressure of 120–180 mmHg, needing a Dacron mesh support to perfuse them [10]. Other studies improved the mechanical properties by drying and crosslinking the collagen with genipin [11], or with plastic compression [12], achieving burst pressures of 1300 and 1600 mmHg, respectively. However, they could not incorporate vascular cell types at the same time as TEBV development, therefore requiring additional steps. Although we obtained low mechanical properties compared to native arteries and veins [24], they were enough to withstand physiological flow rate with shear stress of 10 dynes/cm2, which is similar to shear stress used in previous studies with similar TEBVs sizes [25,26]. Moreover, it is described in the literature that mechanical properties of collagen TEBVs are improved during perfusion time due to extracellular matrix deposition by cells [12,27]. These positive results present the possibility to perfuse vascular cell loaded TEBV and perform mechanical and functional assays.

Regarding HUVEC and HASMCs encapsulation, there was the concern whether these cells would be able to survive and lengthen within the high concentrations of collagen in the ECM, and also whether they would be able to withstand the shear stress produced by the extrusion method. Interestingly, after 24 h of extrusion and culture, viability was 86.1% and 85.8% for 10 and 20 mg/mL, respectively (Figure 2A). These are positive results, as it has been previously described that, generally, viability of cells using microextrusion bioprinting is between 40 and 86% [28,29]. Other studies reported higher viability of other cell types using high collagen concentrations for other purposes. For instance, Lee et al. obtained a viability of approximately 96% using 24 mg/mL of collagen, although the study used cardiomyocytes, assessing the viability only after 1 h of printing [19]. Another study reported viability of approximately 90% using ECs and liver cells (HepG2), up to day 5 of culture [30]. More recently, other authors reported cell viability of NIH 3T3 to be 97, 95 and 87% with 20, 30 and 40 mg/mL after 7 days culture [31]. Nonetheless, our results indicate that the extrusion method is not harmful for these cells and cells could survive in both collagen concentrations. Moreover, when 20 mg/mL of collagen was used, spreading of cells was observed as early as 2 h (Figure 2B), and practically all cells were stretched within 2 days (Figure 2C). Furthermore, TEBV folding was observed at day 5 (Figure 2D), probably due to the strength of cells when adhering and spreading, suggesting a proper environment for their adhesion and proliferation. Therefore, 20 mg/mL of collagen demonstrated to be appropriate for cell viability, and was subsequently used for the next experiments as it would provide more mechanical stability due to the presence of more collagen fibers compared to 10 mg/mL.

Remarkably, with specific HUVEC and HASMC staining with cell trackers, we could confirm that cells were distributed along the entire tubular structure (Figure 3). We also observed the presence of some HASMCs in HUVEC’s inner layer, and vice versa, few HASMCs in HUVEC’s layer. Just after TEBV extrusion, there is the possibility that during the transition of collagen containing cells from a liquid to gel state (around 10 to 15 min), some cells might have moved due to gravity, but not all of them due to the high collagen viscosity. Therefore, some HASMCs and HUVEC might have been in contact with its adjacent layer. Although some cells could not be allocated in their respective layers, that might not be considered a bad outcome. Evidence of cells’ self-organization ability has been previously published [32,33,34]. More specifically, in the vascular field, a previous study reported the extrusion of endothelial cells (ECs) and smooth muscle cells (SMCs) in the same layer, demonstrating that these cells were able to self-organize when perfused [35]. In more detail, ECs could form an endothelium with the presence of tight junctions in the inner lumen core, whereas SMCs were allocated in the adjacent layer. Taking into account these previous reports, with the perfusion of our TEBVs, we would also expect self-organization triggered by flow rate and shear stress stimulus.

## 4. Conclusions

In this study, we were able to directly extrude collagen TEBV structure without structural support, previously reported to be necessary to print collagen structures with lower or similar concentrations. Moreover, vascular cells were able to survive through the manufacturing process and even with high concentrations. Strikingly, 20 mg/mL collagen TEBV possessed enough mechanical properties to withstand physiological flow rates equivalent to arterial shear stress, presenting the possibility to perfuse them to induce their maturation and functionality.

## 5. Materials and Methods

### 5.1. Concentrated Collagen Solution

High concentrated collagen solution is needed in order to obtain a high viscous solution with less probability of spreading when being extruded. Based on previous well-established protocols [36], collagen type I was isolated from bovine tendons obtained from an abattoir. Briefly, tendon was cut to small pieces after being separated from the surrounding fascia. After three washes with phosphate buffered saline (PBS), the small tendon pieces were dissolved with 1M acetic acid for 72 h on agitation. Thereafter, the addition of porcine gastric mucosa pepsin (Sigma-Aldrich, Darmstadt, Germany) was performed for tendon enzymatic digestion at 40 U/mg of tendon under stirring for 2 h at room temperature (<20 °C) and 72 h at 4 °C. To obtain pepsin soluble collagen, the resultant digestion was filtered and purified using 0.9 M NaCl (salt precipitation) for 24 h. Next, 1 M acetic acid was added under constant stirring to the precipitated collagen for 5 days. As a final step, collagen solution was repeatedly dialyzed (MW 8.000 cut off) against 1 mM acetic acid, and finally stored at 4 °C. Collagen purity was assessed using sodium dodecyl sulfate polyacrylamide gel electrophoresis (SDS-PAGE), followed by Coomassie staining and densitometry analysis with ImageJ software.

Once collagen was isolated and its purity was assessed, it was then frozen at −20 °C for at least 24 h, followed by lyophilization (Cryodos-80, Telstar, Andover, ME, USA). Afterwards, lyophilized collagen was cut into small pieces and dissolved to the desired concentration with ice cold 50 mM acetic acid. The concentrations used were 10 and 20 mg/mL.

### 5.2. Support Bath Preparation

For the extrusion of TEBV, we aimed to test two different support baths: (1) gelatin microparticles, and (2) cell culture media alone (endothelial growth medium-2 bulletkit (EGM-2) (Lonza, Basel, Switzerland)). The purpose of testing gelatin microparticles slurry as a support bath was to ensure structural support to the extruded collagen, allowing its gelification without spreading. We also wanted to test if collagen could maintain its structure once extruded into EGM-2 medium without the need for structural support, as there is previous evidence that this might be possible [30].

Gelatin microparticle formation was developed following a previously published method [14]. First, a solution containing a final concentration of 2% (*w/v*) gelatin Type B (Sigma-Aldrich, Darmstadt, Germany), 0.25% (*w/v*) Pluronic F-127 (Sigma-Aldrich, Darmstadt, Germany) and 0.1% (*w/v*) gum arabic (Sigma-Aldrich, Darmstadt, Germany) in 1 L of 50% (*v/v*) ethanol (PanReac AppliChem, Schaffhausen, Switzerland) was heated to 45 °C. The pH was adjusted to 6.25 with 1 M hydrochloric acid (HCl). Then, the beaker was placed on stirrer (MS-H-S, Dlab), sealed with parafilm to avoid solution evaporation and cooled at room temperature while stirring overnight for microparticle formation. To compact the gelatin microparticles, the slurry was divided with 50 mL conical tubes and centrifuged at 300 g for 5 min. After removing the supernatant, the microparticles were resuspended with 1x phosphate buffered saline (1x PBS) at pH 7.4 (washing solution), and centrifuged at 1000 g for 2 min. This washing step was repeated three times. Then, the gelatin microparticle slurry was kept at 4 °C with 1x PBS in its uncompacted state. Prior to TEBV extrusion, gelatin microparticles were compacted by centrifuging them at 2000 g for 5 min. Supernatant was removed and gelatin slurry was transferred into a petri dish.

### 5.3. Collagen Tissue-Engineered Blood Vessel (TEBV) Development

**Figure 4:**
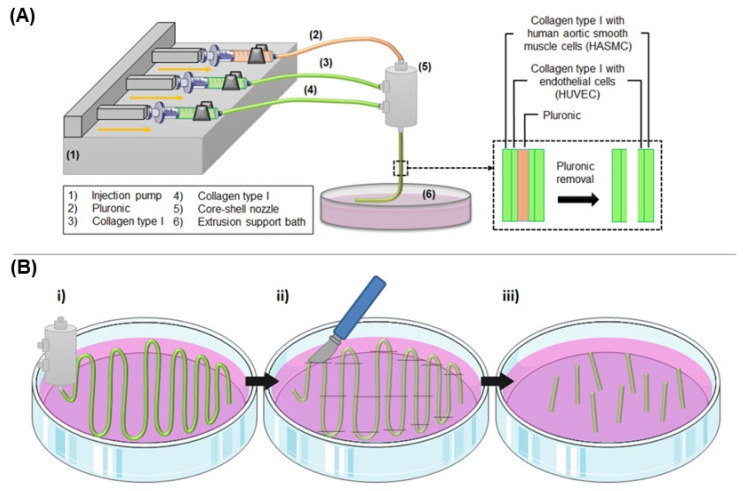
Schematic representation of collagen TEBV development through the triple concentric extrusion method. (**A**) Pluronic (2) and collagen type I (3 and 4) are injected into a triple concentric nozzle (5) in the inner, middle and outer layer, respectively. These biomaterials are extruded in a constant injection speed controlled by an injection pump (1) into a petri dish containing an extrusion support bath or support-free bath (6). Independently of the extrusion support bath, the fibers are extruded at room temperature (approximately 20 °C) for around 5–10 min, followed by incubation at 37 °C for 10–15 min to allow collagen gelation. Then, Pluronic is removed from the inner core by dissolution with cell culture media, obtaining a hollowed dual layer tubular structure. (**B**) (i) Co-axial nozzle extrudes following a zig-zag pattern, and once TEBVs are gelled (ii–iii) the straight parts are cut to the desired length.

Schematic representation of the method is shown in Figure 4. To allow the formation of tricentric fiber, two different materials were injected through a triple concentric nozzle (23 G inner, 17 G middle and 13 G outer) (NanoNC) with the help of injection pumps. The inner syringe was loaded with 25% wt Pluronic F-127 (Sigma-Aldrich) aqueous solution as a sacrificial polymer. The middle and outer syringes were loaded with the previously extracted collagen type I at a concentration of either 10 or 20 mg/mL. Collagen was self-assembled by adding 100 µL of 10x DMEM (Sigma-Aldrich) per each 1 mL of collagen type I solution, and the necessary amount of NaOH, 1 M or 5 M, to neutralize the pH. The solutions were extruded at the same time in a petri dish containing one of the two extrusion baths previously described: gelatin microparticles slurry (at room temperature, approximately 20 °C), or endothelial growth medium-2 (EGM-2, Lonza) at 37 °C.

Three injection speeds were tested (10, 25 and 50 mL/h) to allow the effect of the injection speed on the general parameters of the blood vessel-like structure, mainly shell thickness and inner and outer diameter, to be studied. Besides the effect on the size of the vessel, speed also played a significant role in allowing the deposition of the TEBV at an adequate rate that allows manipulation and proper movement (see Figure 4Bi for the extrusion pattern followed). In this sense, future perspectives aim at allowing the 3D printing of the TEBV, and hence this optimum speed will be required for the printing process. Hence, high speeds may limit this processing aspect, together with an increased shear stress which may ultimately limit cell viability. All the loaded syringes were kept at 4 °C. After 10 min of the extrusion, the petri dish was placed at 37 °C, which further allowed collagen gelification. Afterwards, TEBVs were cut to the desired length (Figure 4Bii–iii), and Pluronic was removed from the inner core by dissolution. To this purpose, TEBVs made from one or the other method were placed in a petri dish with cell culture media during 5 min, and then, cell culture media was flushed through it to remove the rest of the Pluronic, obtaining a hollowed TEBV structure. To assess the feasibility of perfusion of TEBVs, they were cut to 2.5 cm length, the appropriate size to incorporate them in the perfusion chamber. For cell viability and 3D reconstruction assays, TEBV were cut to a length of 1 cm.

### 5.4. Burst Pressure

Burst pressure is defined as the maximum pressure that a TEBV can withstand before rupture. In order to measure it, TEBVs with the highest concentration (20 mg/mL) of collagen were attached in the perfusion chambers with one end sealed and the other connected to a differential pressure gauge (Leo2, Keller, London, UK). PBS was injected through TEBVs until failure, and the maximum pressure achieved was recorded. Burst pressure was performed with cell-free TEBVs to record the basal mechanical properties. A total of three TEBVs were analyzed.

### 5.5. Proof of Concept of TEBV Perfusion

After measuring the burst pressure of the TEBVs, we wanted to check if these TEBVs were able to withstand physiological flow rate with an arterial shear stress of 10 dynes/cm^2^ to perform functional assays in the future. To this purpose, we calculated the appropriate flow rate to provide a shear stress of 10 dynes/cm^2^ according to our TEBVs size with Poiseuille formula: $SS=\frac{4µQ}{\pi r^{3}}$, where *SS* is the shear stress, *µ* is the viscosity of the fluid, *Q* is the fluid rate, and *r* is the radius of the vessel. As a proof of concept, we tested if they could withstand physiological arterial shear stress with cell free TEBV containing collagen at a concentration of 20 mg/mL and perfuse with endothelial cell growth medium-2 (EGM-2, Lonza). The perfusion system consisted of a closed tubing loop which connected the cell culture media reservoir with the perfusion chamber containing the TEBVs. The flow rate was achieved by using a peristaltic pump (Masterflex L/S Standard Digital Drive, Cole Parmer, Vernon Hills, IL, USA) connected with a head pump (Masterflex L/S easy load head pump, Cole Parmer).

### 5.6. Cell Culture

For the encapsulation of vascular cells within TEBV structures, human umbilical vein endothelial cells (HUVECs) (Lonza) and human aortic smooth muscle cells (HASMCs) (ATCC) were used. For their expansion, HUVEC were seeded with endothelial growth medium-2 bulletkit (EGM-2) (Lonza) at a cell density of 2500 cells/cm^2^. HASMCs were seeded with vascular cell basal medium (ATCC) supplemented with vascular smooth muscle cell growth kit (ATCC) at a cell density of 2500 cells/cm^2^. Both cell types were passaged using 0.25% Trypsin-EDTA (Gibco, St. Louis, MO, USA) when they reached 70–85% confluence. Both cell types were cultured at 37 °C and 5% CO_2_.

For cell encapsulation, the procedure described in Section 2.3 was followed, incorporating HUVEC cells at a concentration of 15 × 10^6^ cells/mL in the middle collagen syringe, and HASMCs at a concentration of 10 × 10^6^ cells/mL in the outer collagen syringe.

### 5.7. Cell Viability Assay

Our initial hypothesis is that the increase of collagen concentration increases the stability of the TEBV. However, there is uncertainty whether encapsulated cells would be able to survive with higher concentrations and the effect of the shear stress produced by the extrusion method. For this reason, we aimed to assess the viability of HUVEC and HASMCs when encapsulated within TEBVs with two collagen concentrations: 10 and 20 mg/mL. To this purpose, a LIVE/DEAD cell imaging kit was used following the manufacturer’s instructions (Invitrogen). Briefly, live cells were stained with a non-fluorescent cell-permeant component (Calcein AM) that was converted by intracellular enzymes to fluorescence Calcein AM, giving a green fluorescence signal at 488/515 nm (excitation/emission wavelengths), and dead cells were stained with a cell-impermeant component (BOBO-3 iodide) as it enters within cells with a compromised cell membrane, producing a red nuclear fluorescence at 570/602 nm (excitation/emission wavelengths). Both components were added at equal volumes in the cell culture medium with TEBVs, with a final concentration of 1x. Cells were imaged with a laser scanning confocal microscope (Leica SP8, LAS X software version 3.5.5.19976) after 15 min of incubation in the dark. For the viability analysis, three samples per time point were analyzed, and three captures at different plans per sample were acquired. Live and dead cells were counted from each image using ImageJ software (ImageJ 1.52 a). Viability was calculated as a percentage, as the number of live cells divided per total number of live and dead cells. The viability results determined which concentration of collagen was used for the subsequent experiments.

### 5.8. Reconstruction of TEBV with Fluorescent Labeled HUVECs and HASMCs

In order to check if HUVEC and HASMCs could remain in their respective layers once extruded, inner and outer layers, respectively, each cell type was stained with different colored cell tracker fluorescence probes prior to the development of TEBV structures. HUVEC cells were stained with Green CMFDA (Invitrogen, Waltham, MA, USA) and HASMC were stained with Red CMTPX (Invitrogen), emitting a green and red signal, respectively. The final concentration in the cell culture media of both cell trackers was 10 µM. After adding each cell tracker into its respective cell culture media, cells were incubated with them for 45 min at 37 °C, while protected from light. For blood vessel formation, cells were added at each specific syringe, as explained in Section 5.3 and Section 5.6. TEBVs were imaged at day 2 using a confocal laser microscope (Leica SP8, LAS X software version 3.5.5.19976). The excitation/emission wavelengths were 492/517 for Green CMFDA and 577/602 for Red CMTPX. Images were processed and 3D reconstruction was performed with the same confocal software.

### 5.9. Statistical Analysis

Statistical analysis was performed using SPSS software (SPSS v21, IBM). Kruskal–Wallis and Mann–Whitney U non-parametric tests were used to compare if there were differences between the outer diameter, inner diameter and wall thickness of TEBVs extruded with two different collagen concentrations for each injection speed. Data was represented as mean ± standard deviation; *n* = 8. A *p*-value of less than 0.05 was considered statistically significant.

## Data Availability

Data is contained within the article or supplementary material.

## Supplementary Materials

The following supporting information can be downloaded at: https://www.mdpi.com/article/10.3390/ijms23105618/s1.
