# Supplementary figures and images for: Biofabrication of Collagen Tissue-Engineered Blood Vessels with Direct Co-Axial Extrusion
Source: Int J Mol Sci. 2022 May 17;23(10):5618. doi: 10.3390/ijms23105618 (PMC9144639; doi:10.3390/ijms23105618)

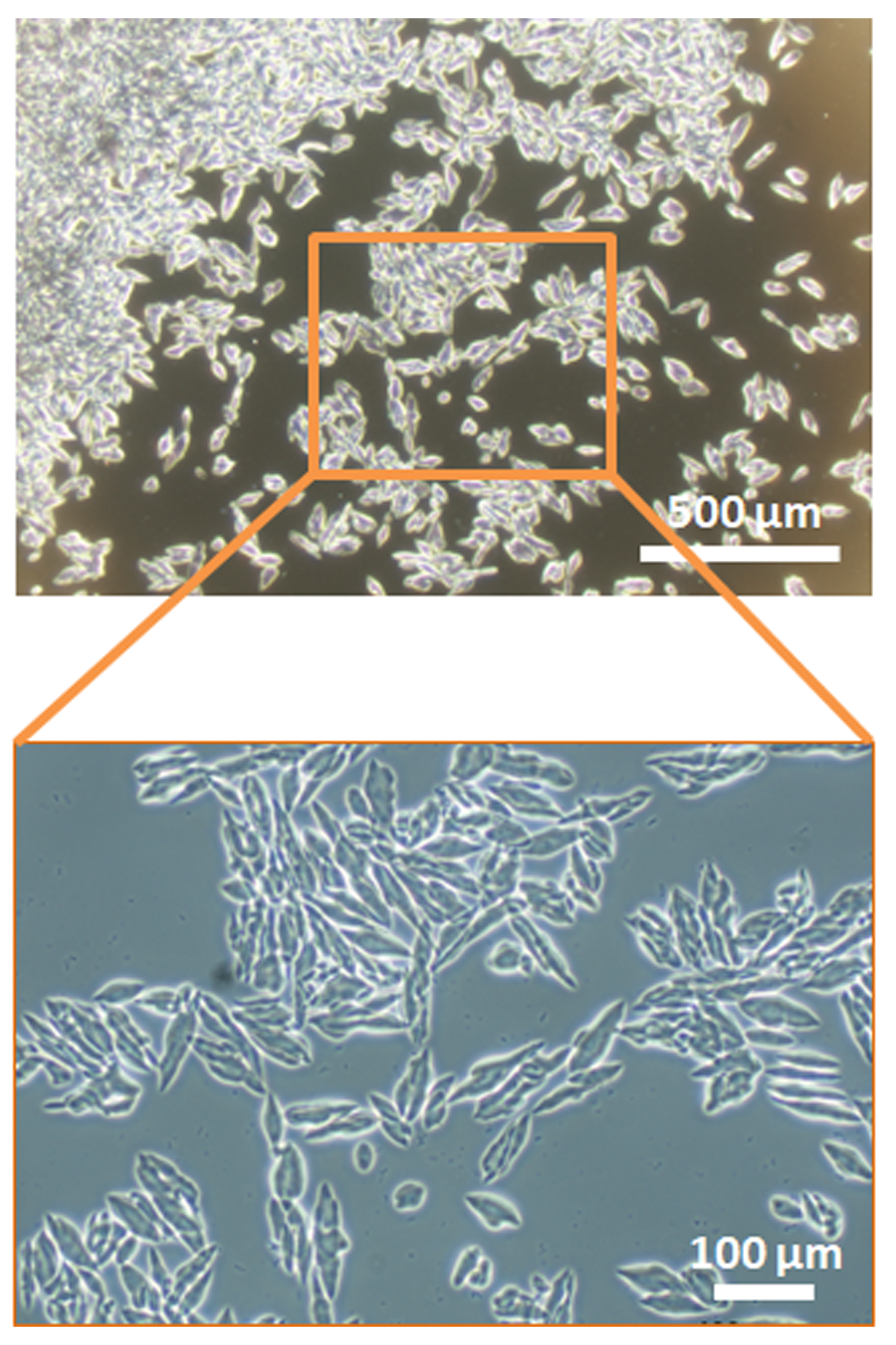

Supplement: Supplementary file 1 [file ijms-23-05618-s001.zip › Supplementary Figure S1.tif]
